# Molecular Basis of Plant–Pathogen Interactions in the Agricultural Context

**DOI:** 10.3390/biology13060421

**Published:** 2024-06-06

**Authors:** Usman Ijaz, Chenchen Zhao, Sergey Shabala, Meixue Zhou

**Affiliations:** 1 Tasmanian Institute of Agriculture, University of Tasmania, Launceston, TAS 7250, Australia; usman.ijaz@utas.edu.au (U.I.); chenchen.zhao@utas.edu.au (C.Z.); 2 School of Biological Science, University of Western Australia, Crawley, WA 6009, Australia; sergey.shabala@uwa.edu.au; 3 International Research Centre for Environmental Membrane Biology, Foshan University, Foshan 528000, China

**Keywords:** agriculture, climate change, pathogens, resistance mechanisms, genome engineering

## Abstract

**Simple Summary:**

Plants encounter numerous biotic and abiotic challenges during their life cycle. Biotic stressors pose serious threats to crop yield, causing food security issues. Different signaling pathways such as recognition receptors help to recognize pathogen invasion and activate the plant defense response. Understanding the plant–pathogen interaction at a molecular level is crucial for developing strategies to enhance resistance and to mitigate the impact of plant diseases on agriculture productivity.

**Abstract:**

Biotic stressors pose significant threats to crop yield, jeopardizing food security and resulting in losses of over USD 220 billion per year by the agriculture industry. Plants activate innate defense mechanisms upon pathogen perception and invasion. The plant immune response comprises numerous concerted steps, including the recognition of invading pathogens, signal transduction, and activation of defensive pathways. However, pathogens have evolved various structures to evade plant immunity. Given these facts, genetic improvements to plants are required for sustainable disease management to ensure global food security. Advanced genetic technologies have offered new opportunities to revolutionize and boost plant disease resistance against devastating pathogens. Furthermore, targeting susceptibility (S) genes, such as *OsERF922* and *BnWRKY70*, through CRISPR methodologies offers novel avenues for disrupting the molecular compatibility of pathogens and for introducing durable resistance against them in plants. Here, we provide a critical overview of advances in understanding disease resistance mechanisms. The review also critically examines management strategies under challenging environmental conditions and *R*-gene-based plant genome-engineering systems intending to enhance plant responses against emerging pathogens. This work underscores the transformative potential of modern genetic engineering practices in revolutionizing plant health and crop disease management while emphasizing the importance of responsible application to ensure sustainable and resilient agricultural systems.

## 1. Introduction

The world population is currently increasing by approximately 1.1% per year [1], and if this trend continues, the global population is projected to reach 9.9 billion by 2050 [2]. Consequently, global food production needs to increase by 70% to effectively meet the nutritional requirements of the growing population [3]. At the same time, climate-driven constraints threaten crop production worldwide [4]. At least half of the losses in agricultural production systems are due to biotic stresses imposed by pathogenic fungi, bacteria, viruses, and nematodes [5]. In some cases, new races of pests and diseases have resulted in global yield losses ranging from 10% to 40% [6], impacting the agriculture industry with annual losses exceeding USD 220 billion [7]. For instance, emerging strains of wheat blast [8,9] and the wheat stem-rust fungus Ug99 [10] reduced wheat production by 15% in 2017 and 2018. Usually, chemical fungicides and pesticides are extensively used to control plant diseases, which has resulted in environmental pollution, harmful impacts on beneficial microorganisms, and the emergence of new pathogens [11]. Therefore, urgent efforts are required for the sustainable development of the agriculture system to address biotic stresses and to increase food production.

Plant disease resistance is usually categorized into major resistance (qualitative) and partial resistance (quantitative) [12,13]. Major resistance in plants depends on the presence of resistance genes (*R*), which encode intracellular immune receptors such as nucleotide-binding leucine-rich repeats (NLRs) and immune receptors like receptor-like kinases. These cellular immune receptors detect specific avirulence (Avr) proteins/cognate effectors or conserved pathogenic molecules either directly or indirectly [14]. Consequently, most *R* genes confer race-specific resistance against pathogens that can be easily disrupted, as pathogens rapidly evolve to mutate the cognate *Avr* genes to escape host recognition [15]. In contrast, partial resistance is controlled by quantitative trait loci, which provide long-term resistance against pathogens [16]. Crop improvement programs based on plant resistance genes/QTLs need to be optimized and require more in-depth studies to overcome the existing challenges posed by phytopathogens. The only feasible approach to address this alarming situation is to deploy innovative and advanced strategies or to identify resistance mechanisms to combat biotic stresses. In recent decades, advancements in biotechnology approaches have facilitated the understanding of plant–pathogen interactions and explored the physiological and molecular defense mechanisms in plants that confer resistance against pathogens. For example, it was reported that the plant growth hormone salicylic acid (SA) activates systemic acquired resistance and regulates stomatal closure via the cell-specific transcription factor NTM1-LIKE 9 (NTL9) upon pathogen attack [17,18]. However, despite this progress in understanding disease resistance mechanisms, the fine print of plant–pathogen interactions in a molecular context remains incomplete. Exploring these gaps is crucial to further inform modern genetic engineering approaches to improving crop disease resistance in agricultural systems.

The rapid expansion of genome-engineering techniques has enabled the investigation of the molecular basis of plant–pathogen interactions. Genome-engineering approaches have allowed us to explore new functions or to enhance existing ones in crop plants [19,20]. Recently, targeting *R*-gene-mediated pathogen resistance using CRISPR/Cas9 has provided high-yield crop varieties and durable disease resistance against pathogens [21]. Liu et al. [22] reported that knocking out the *GmTAP1* susceptibility gene in *Glycine max* enhanced resistance against *Phytophthora sojae* compared with wild-type plants. In another study, Zhou et al. [23] created targeted mutations in the *ERF922* gene and observed strong resistance against bacterial blight disease in mutant rice plants compared with non-mutated plants. In this regard, it is expected that *R*-gene-based genome engineering will be more powerful, cost-effective, and rapid than natural evolution. In this article, we summarize the progress made in the current understanding of pathogen resistance mechanisms, their implications for the agriculture sector, and the prospects of CRISPR-mediated genome engineering of *R* genes to improve plant resistance against different pathogens. Moreover, new strategies for improving durable disease resistance in crops against emerging pathogens are also proposed.

## 2. Host-Based Factors Affecting Pathogen Intrusion

### 2.1. Immune Attenuation

The distribution of energy between defense and growth requires constant fine-tuning of the signaling process, including the immediate attenuation and activation of processes that significantly overlap. Rapid activation and engagement of broad immune signaling processes, such as those involving mitogen-activated protein kinases (MAPKs), are required against pathogen resistance following pathogen perception [24,25]. The MAPK cascade is one of the best-known immune signaling systems in activating the plant immune response against pathogen attack. In *Arabidopsis thaliana*, signaling facilitated by MAPK3/6, which triggers its inhibitory process, provides a clear illustration of immune attenuation [26]. Phosphorylation of MKP1 by MAPK6 was saturated for approximately 10 min after pathogen-associated molecular pattern (PAMP) treatment, leading to the stabilization and elevation of MAPK1 protein levels [27] Besides MAPKs, MKPs might dephosphorylate various substrates involved in immune signaling, as evidenced by the fact that MKP1 regulates thousands of MKP6-independent pro-immune transcription reactions within 90 min following flg22 elicitation in *A. thaliana* [28]. Furthermore, in line with MKP1’s function as a broad regulator of immune attenuation, previous studies have shown that MKP1 acts as a negative regulator in the defense response against hemibiotrophic pathogens (e.g., *Pseudomonas syringae*) in *A. thaliana*, and biotrophic pathogens (e.g., *Ralstonia solanacearum*) in tomato [29].

Other than MKPs, protein phosphatases (i.e., PP2A/Cs) play a substantial role in reducing kinase activity during immune signaling, contributing to immune attenuation [30]. For instance, Jagodzik et al. [31] reported that groups of PP2Cs such as Hal1/2/3 interact with MAPK3/6 and inactivate flg22-mediated jasmonic acid (JA) signaling, a crucial virulence mechanism used by pathogens to control immune signaling in plants. The dephosphorylation of MAPK4/6 by Arabidopsis AP2C1 modulates SA- and JA-associated immune signaling. In another study, Guo et al. [32] reported that the protein phosphatase GhAP2C1 interacts with GhMPK4 and attenuates the immune response to enhance resistance against *Fusarium oxysporum* in cotton. Other kinases, such as the pattern-triggered immunity (PTI) signaling regulators BAK1, CPK6, and BIK1, also modulate the activation of PP2A/Cs under biotic stress in plants [33]. Further exploration of the complex network of molecular responses mediated by the plant immune attenuation system can help to further elucidate these mechanisms for developing robust strategies for long-term resistance against pathogens.

### 2.2. Developmental Stage

Plants experience dynamic changes in abiotic and biotic conditions during their life cycle, developing unique ecological niches for host–plant interactions. Plant phenology is strongly correlated with the strength of the plant’s immune system [34,35]. Immune signaling becomes more robust from the early developmental stage to the reproductive period; thereafter, the strength of the immune system decreases due to host senescence [36]. A recent eco-genetic study showed two approaches adopted by Arabidopsis plants to enhance pathogen resistance: shortening of the vegetative stage to promote fast reproduction to prevent pathogen infection and death, or prolongation of the vegetative stage to obtain more energy for the development of a robust immune system [35,37]. The strength of the immune system increases from early- to late-developed organs due to the spatial scale process. For example, when rice plants were infected with *Xanthomonas oryzae* pv. *Oryzae*, mild disease symptoms were observed in adult leaves compared with juvenile leaves [38]. In another study, cauline leaves, adult rosettes, and juvenile rosettes from 8-week-old Arabidopsis plants showed variable resistance against *Sclerotinia sclerotiorum* [39]. Similar results were also observed in tobacco plants inoculated with *S. sclerotiorum*.

Moreover, the age-related robustness of the immune system depends on the functions of essential phytohormone signaling components, as evidenced by decreased resistance in cauline and adult leaves of the hormone biosynthesis mutants *abab1* (abscisic acid), *jar1* (jasmonic acid), and *sid1* (salicylic acid) compared with control plants [40]. However, the comprehensive molecular basis of developmental-stage-associated host resistance is unclear, and further studies are required to explore the underlying mechanisms of developmental-stage-associated host resistance against pathogens.

## 3. Underlying Mechanisms to Counter Pathogen Attack

### 3.1. Physiological Mechanisms

#### 3.1.1. Phytohormones

**Figure 1:**
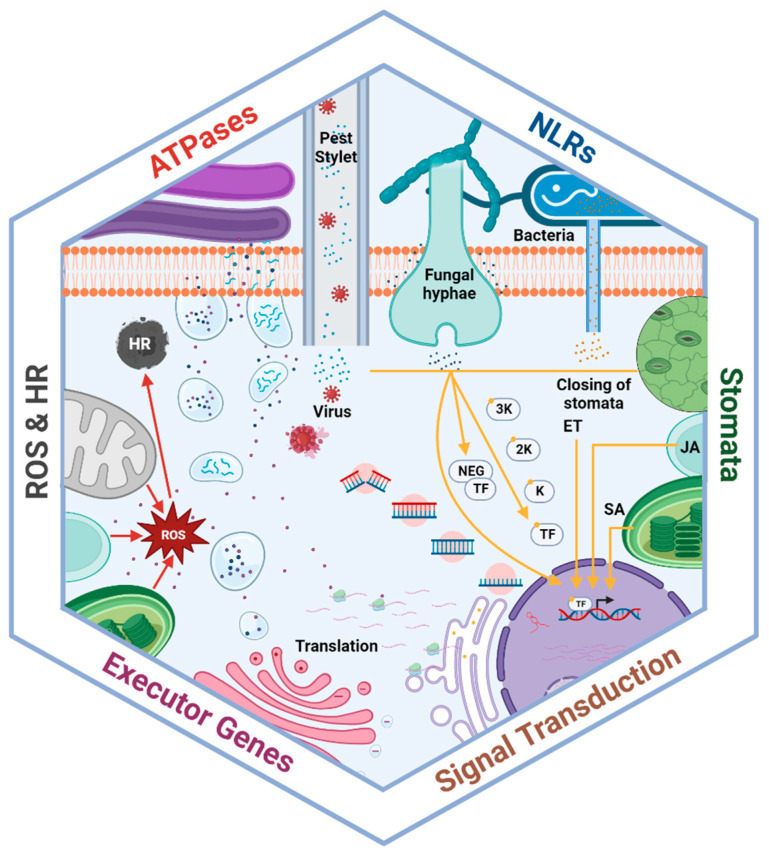
Components of plant disease resistance mechanisms involved in pathogen detection, signal transduction, and the defense response. Effectors produced by bacteria, fungi, and viruses stimulate plant receptors to activate signaling cascades. Activated receptors then directly function as transcription factors or activate signaling pathways, including for hormone production, the hypersensitive response, reactive oxygen species production, and genes involved in the defense response. These defense-related compounds actively inhibit pathogen intrusion through stomatal closure and/or make pathogen infection more difficult. Moreover, other organelles further improve the defense response, including peroxisomes and chloroplast, which produce phytohormones, as well as the nucleus, Golgi apparatus, and endoplasmic reticulum, which trigger antimicrobial protein production.

Plants produce a diverse array of metabolites that are crucial for activating defense signaling against phytopathogen attacks (Figure 1). Among these metabolites, ABA, ethylene (ET), JA, and SA play important roles and are responsible for mediating defense responses in pathogen life cycles [41,42]. SA regulates local and systemic resistance responses to biotrophic and hemibiotrophic pathogens and interacts with numerous developmental and response functions [43]. SA and MAPK cascades can act upstream of each other, with SA activating MAPK cascades and some cascades stimulating SA activity [44,45]. SA transfers signals regarding pathogen presence through a complex signaling cascade, activating transcription factors to induce the expression of defense-related genes. After initiating a signaling cascade, SA mediates the reduction of disulfide bonds within the oligomeric nonexpressor of pathogenesis-related genes 1 (NPR1) protein by thioredoxins. This reduction process facilitates the translocation of NPR1 monomers from the cytosol into the nucleus. Within the nucleus, these monomers bind to the transcription factor TGA, specifically at the TGACG binding site, and upregulate the expression of resistance genes [46,47].

ET and JA are essential for conferring resistance against herbivorous insects and necrotrophic pathogens. These hormones stimulate the secretion of volatile compounds in response to caterpillar oral secretions [48]. The synthesis of ET increases in response to the detection of bacterial flagellin [49]. ET inactivates the constitutive triple response1 (CTR1) protein and its receptors, reducing EIN2 and EIN3 expression and allowing the upregulation of ET signaling and necrotroph resistance [50,51]. Pathogen interference also targets this pathway, with the XopD effector from *Xanthomonas euvesicatoria* bacterium desumoylating the transcription factor SIERF4 to disrupt hormone signaling, particularly inhibiting ET synthesis and resistance [52]. In addition to ET and JA, auxin and ABA are involved in plant resistance against pathogens [53]. Systemic acquired resistance may also rely on the salicylic acid signaling pathway, as shown in experiments on watermelon’s responses to *F.oxysporum* infection [54]. Peptides can also act as plant hormones. Some studies have demonstrated that small peptide hormones like systemin are involved in the systemic herbivory response, resulting in changes in gene expression, particularly in neighboring plants not exposed to biotic stress [55,56]. This suggests that plant hormones can facilitate communication among individual plants to enhance resistance.

#### 3.1.2. Stomatal Immunity

Plants have developed sophisticated mechanisms to recognize microbial infections and prevent pathogen intrusion through controlled stomatal regulation. Numerous studies have investigated the role of the stomata in plant innate immunity in pathogen resistance [57,58]. PAMPs induce stomatal closure within 1 h after pathogen recognition by host cells [59]. For example, wheat plants rapidly close their stomata upon detecting bacterial molecules via the FLS2 (flagellin-sensing 2) receptor [60] Similarly, the application of elicitors such as chitosan or oligogalacturonic acid, associated with fungal invasion, stimulates stomatal closure in the Asiatic dayflower and tomato under biotic stress conditions [61], while syringomycin, a bacterial phytotoxin, induces stomatal closure in broad bean [62].

Moreover, stomatal closure is regulated by various pathways involving functional and regulatory components, including receptors, ion channels, secondary messengers, phosphatases, and protein kinases [63]. The elevated levels of SA following pathogen invasion stimulate the generation of secondary messenger ions such as Ca^2+^, NO, and reactive oxygen species (ROS) [64]. These secondary ion messengers further activate the SLAC1 (anion) and GORK ion channels to facilitate stomatal closure [65]. Moreover, abscisic acid (ABA) plays a vital role in promoting stomatal closure during pathogen invasion [53].

However, prolonged stomatal closure can reduce transpiration and photosynthesis, creating an aqueous apoplast that promotes pathogen colonization. Plant genomes encode thousands of peptides that regulate reproduction, development, and long- and short-distance signaling in plants [66,67]. Peptides such as PLANT SCREW UNRESPONSIVE RECEPTOR (NUT) and SMALL PHYTOCYTOKINES REGULATING DEFENSE AND WATER LOSS (SCREWs) secreted by plants regulate PAMP- and ABA-induced stomatal closure. SCREWs, sensed by NUT, function as immunoregulatory phytocytokines and recruit the SOMATIC EMBRYOGENESIS RECEPTOR-LIKE KINASE (SERK) co-receptor to modulate immune signaling. SCREWs stimulate the NUT-dependent phosphorylation of ABI1 and ABI2, resulting in reduced activity of S-type ion channels and increased activity of ABI1 toward PAMP- and OST1-induced stomatal closure. Following initiation by pathogen infection and dehydration, SCREW-NUT signaling promotes apoplastic water loss and disrupts the pathogen-rich aqueous environment to prevent colonization [68].

#### 3.1.3. Hypersensitive Response, Reactive Oxygen Species Burst, and Cell-Wall Modifications

The hypersensitive response (HR) is the most common immune response in plants, inducing programmed cell death (PCD) at the site of pathogen infection to prevent the further spread of infection to healthy tissues. Pathogen infection stimulates peroxidase activity, leading to the production of ROS [26,69]. NADPH oxidases, particularly RBOHD, play a main role in this process by triggering a burst in apoplastic H_2_O_2_ accumulation. RBOHD interacts with pattern-recognition receptors (PRRs) and phosphorylates BIK1 to activate ROS production [70]. ROS stimulates PCD, and H_2_O_2_ spreads to adjacent cells to initiate the production of chemicals such as glutathione to prevent oxidative damage [71,72]. In addition to triggering HR, ROS creates an unfavorable environment for pathogen reproduction and survival [73]. Oxalate oxidases and amines are also involved in ROS generation, along with NADPH oxidases and peroxidases [74]. Barley and wheat produce oxalate oxidases, known as germins, which break down oxalic acid secreted by pathogens to enhance resistance. Transgenic plants with barley and wheat oxidase genes have shown improved resistance to *S. sclerotiorum* compared with control plants [75,76].

Pathogens are required to overcome physical barriers and penetrate the cell wall to enter the host plant for successful infection. The plant cell wall is reinforced with various polymers at sites of pathogen attack to inhibit intrusion [77]. Silicon (Si) is the most observed polymer, along with lignin, callose, and extensins, deposited in the cell wall to prevent pathogen infection [78]. This deposition is supported by the upward translocation of Si through the apoplast, followed by Si deposition in the extracellular spaces of xylem vessels and leaf epidermal cells [79]. This process forms a thick layer of the Si–cuticle complex in the membrane, providing mechanical resistance and enhancing plant resilience against pathogen attack. Si also forms complexes with organic compounds in epidermal cell walls, providing further strength and reducing the disease intensity [80]. Likewise, Abdelrhim et al. [81] reported that the application of Si improves resistance against *Rhizoctonia solani* in wheat plants by increasing extracellular adsorption in the spongy mesophyll and air spaces of the leaf. Si application also improved the resistance against *Fusarium* wilt by promoting cell-wall modification and the antioxidative potential [82].

### 3.2. Molecular Mechanisms Underpinning R Genes

Host reprogramming by the passive loss of susceptibility mutations in cellular pathways represents a common strategy that offers durable resistance against various pathogens. This form of susceptibility loss is typically governed by recessive traits, but sometimes, dominant alleles are also involved. Genes associated with this phenomenon are termed adult plant resistance (*APR*) genes, as they confer resistance to plants at the adult stage [83]. Moreover, *APR* genes commonly provide partial protection against a broad spectrum of pathogens [84,85]. Furthermore, loss-of-interaction and perception-based mechanisms specifically function in adult plants. For instance, recessive loss-of-function MLO (Mildew Locus O) alleles serve as key regulators of a senescence-associated loss-of-susceptibility mechanism in both monocots and dicots. MLO encodes a protein integral to membrane function, acting as a negative regulator of cell death under biotic stresses. Spontaneous cell death is associated with MLO loss-of-function alleles [85].

In barley and *A. thaliana*, MLO co-expresses with PEN1/2/3 (or its barley orthologs), which are required to activate the immune response against powdery mildew disease. MLO serves as a negative regulator of the PN1/2/3 pathways, as these genes are essential for MLO-dependent immunity in both *A. thaliana* and barley [86]. Therefore, loss of the general cell-death suppressor confers resistance by deregulating the PEN1/2/3 pathway in both monocots and dicots. This principle has been applied to develop transgene-free tomatoes resistant to powdery mildew disease [87]. Similar to the MLO resistance mechanism, a loss-of-function mutation in the *Pi21* gene, which encodes an HMA-domain protein, functions to inhibit the plant defense response and confers recessive resistance against rice blast [88,89]. Notably, HMA-domain proteins serve as essential virulence targets for several pathogenic fungi. Effectors with unrelated sequences adopt a common fold and integrate with a domain of HMA proteins [90,91].

Therefore, analogous to *pi21*, the generation of loss-of-function alleles from genes encoding HMA domains confers resistance to many pathogenic fungi in different plant species. Similarly, the dominant *R* gene *Lr67* in wheat provides partial resistance to stem rust, leaf rust, and powdery mildew, primarily due to mutations in a hexose transporter that differs from the susceptible allele by two amino acids. The heterodimerization of *Lr67* with the susceptible allele product exhibits dominant negative effects, resulting in decreased glucose uptake and leaf-tip necrosis [92,93]. The resistance conferred by these genes depends on the deregulation of early defense responses.

#### 3.2.1. Disrupting Interaction with Key Host Targets

Disrupting the interaction with key host susceptibility factors is a common mechanism underlying recessive *R* genes. Indeed, various identified *R* genes are involved in loss-of-interaction mechanisms that confer resistance against viruses [94]. Most of the known *R* genes act against potyviruses and encode 4E or 4G family mutant translation initiation factors, which are unable to interact with viral transcript cap structures, thereby imparting resistance to potyviruses [95]. Moreover, about one-third of the protective *R* genes involved in resistance against bacterial blight disease are recessively inherited, and disrupting interaction with key host targets appears to be an underlying mechanism [96]. A recessive mutation in the promoter area of the *xa27* allele prevents TALEAvrXa27 (Transcription activator-like effectors) from being manipulated, resulting in the loss of susceptibility [97]. Similarly, a mutation in a single amino acid of the recessive *xa5* gene encoding IIa OsTFIIAγ5 (gamma subunit of a transcription factor) restricts bacterial movement and confers resistance to different *X. oryzae* strains in the adult plant [98]. OsTFIIAg5 interacts directly with TALEs to complete their activity [99]. The mutation in *Xa5* seems to affect the Xa5-TALEs interaction, thus showing a varying degree of resistance against many pathovars of *X. oryzae* [100,101].

#### 3.2.2. NLR Activation by the Direct Intracellular Recognition of Effectors

The direct recognition of effectors is not limited to the cell surface, as various effectors have been reported to directly interact with nucleotide-binding leucine-rich repeat proteins (NLRs) to stimulate a defense response against pathogens [102]. For example, an effector of Hyaloperonospora Arabidopsis thaliana recognized1 (ATR1) interacts directly with the NLR *Peronospora parasitica*1 (RPP1), resulting in its recognition [103]. Numerous RPP1 alleles are perceived specifically by different ATR1 alleles, and the LRRs of RPP1 mediate this specificity [104]. Similarly, another effector, AVRL567 from *Melampsora lini*, binds directly to its cognate receptors, the L5, L6, and L7 NLRs, which are encoded by allelic genes [105,106]. These receptors recognize AvrL variants differently, with the LRR domain of the NLR determining this specificity [107,108]. Interestingly, cooperative polymorphisms in other NLR domains also influence the perception of effectors, showing a perception mechanism in which intramolecular interactions compete with effector binding. The L5, L6, and L7 effectors exist in “off” and “on” equilibrium states, and the binding of effectors triggers immune signaling by stabilizing them to the “on” state [106,109]. This equilibrium model might have a wider range of applications in NLR signaling. Indeed, 21-amino acid peptides from the viral movement protein (NSm21) are directly recognized by the NLR Sw-5b, and it has been determined that NSm21 binding disrupts the interaction between NB-ARC and the leucine-rich repeat (LRR) domain [110].

Variants of allelic effectors are perceived by allelic NLR-encoding genes in the case of L5/6/7 and RPP1. However, the perception of homologous effector proteins by homologous and/or allelic NLRs is not constant. For instance, barley and rice both have similar NLRs located within the *R* gene cluster. These NLRs can recognize distinct effectors from *Blumeria graminis* and *M. oryzae*, despite the differences in their genetic sequences [111,112]. Moreover, potato orthologs of two distinct NLR-encoding genes in tomato L2, which recognize a fungal effector, and Sw-5b, a viral effector, can also identify effector proteins from oomycetes [113,114]. The exact molecular mechanism by which highly similar NLRs detect sequence-unrelated effectors from different pathogens is still to be unraveled. In several cases, the C-terminal LRR region of the NLR has the specificity-determining region [115,116]. Minor variations across various regions of the NLR appear to prompt its recognition of unrelated effectors. Moreover, the effectors might evolve distinct specificities to sequence-unrelated effectors by adopting a similar fold. Indeed, gaining a structural understanding of how these NLRs bind to effectors could offer more insights into the molecular mechanisms of direct perception [117,118].

#### 3.2.3. Active Loss of Susceptibility

Active loss-of-susceptibility mechanisms encode host proteins that disarm pathogens by disrupting their life cycle. These mechanisms are diverse and provide resistance against various pathogens. The active loss of susceptibility mechanisms triggers the generation of danger-associated molecular patterns (DAMPs) and PAMPs after pathogen recognition [119]. For example, the first cloned *R* gene, *Hm1*, encodes an NADPH-dependent reductase usually involved in detoxifying the HC toxin in maize (Table 1) [120]. Cochliobolus carbonum race 1 (CCR1) is a key virulence factor of HC, causing ear mold and leaf blight in maize. Hm1 orthologs exist in barley and the grass family, and such orthologs play a significant role in providing non-host resistance against CCR1 [117]. Numerous active loss-of-susceptibility mechanisms are employed to develop resistance against viruses in plants. For instance, the *Tm-2* gene product of tomato provides resistance against the tomato mosaic virus and inhibits replication by binding to replication proteins [118]. Furthermore, the resistance genes *Ty-1/Ty-3* in wild tomatoes encode γ-clade RNA-directed RNA polymerases. These enzymes initiate RNA-directed DNA methylation, offering protection against single-stranded DNA geminiviruses [121].

#### 3.2.4. TAL Effector-Dependent Expression of Executor Genes

Executor genes comprise a new class of *R* genes that are transcriptionally activated by TALEs produced by Xanthomonas species, conferring resistance against Xanthomonas strains carrying these TALEs. The TALEs secreted by Xanthomonas species act as important virulent factors that regulate the expression of susceptibility (*S*) genes for disease development [122,123]. Executor genes function as promoter traps, stimulating the transcription of immunity-related genes. The promoter of executor genes serves as a decoy by replicating the promoter regions of susceptibility factors, which activates the defense response [124]. Various executor genes have been identified in and cloned from different plant species as being able to confer resistance against different phytopathogens, such as *Xa10* [125], *Xa23* [126], and *Xa27* [127] in rice and *Bs4C-R* [128] and *Bs3/Bs3-E* [129] in pepper. Previously reported executor genes (*Bs4C-R*, *Xa10*, *Xa23*, and *Xa27*) either encode a protein with many putative transmembrane domains or encode a protein with catalytic activity for flavin monooxygenase (Bs3 and Bs3-E). However, an improved understanding of the specificity of DNA binding to TALEs provides more insights into developing immunity against various Xanthomonas strains [130,131]. This approach was also employed to engineer RipTALs for resistance development against *R. solanacearum* in tomatoes [132].

**Table 1:** List of cloned *R* genes against different diseases in plants.

Crop Specie	Gene	Protein Type	Disease	Pathogen	Reference
Barley	*Stb6*	Receptor kinase	Septoria tritici blotch	*Zymoseptoria tritici*	[133]
	*Mla1*	NB-LRR	Powdery mildew	*Blumeria graminis*	[134]
	*Mla6*	NB-LRR	Powdery mildew	*B. graminis*	[135]
	*Rpg1*	Protein kinase	Stem rust	*Puccinia graminis*	[136]
Wheat	*Pm3*	NB-LRR	Powdery mildew	*B. graminis*	[137]
	*Lr10*	NB-LRR	Leaf rust	*P. triticina*	[55]
	*Lr21*	NB-LRR	Leaf rust	*P. triticina*	[137]
Maize	*ZmTrxh*	H-type thioredoxin	Lethal necrosis	*Sugarcane mosaic* *virus*	[138]
	*Rp1-D*	NB-LRR	Leaf rust	*Puccinia sorghi*	[139]
	*Rxo1*	NB-LRR	Bacterial streak	*Xanthomonas* *oryzae*	[140]
	*Hm1*	HC toxin reductase	Corn leaf blight	*Cochliobolus* *carbonum*	[120]
	*Hm2*	HC toxin reductase	Corn leaf blight	*C. carbonum*	[141]
	*Rp3*	NB-LRR	Leaf rust	*Puccinia sorghi*	[71]
	*qRfg1*	CCT-domain gene	Gibberella stalk rot	*Fusarium* *graminearum*	[142]
	*qMdr9.02*	Lignin biosynthesis	Multiple	Multiple	[143]
Rice	*Piz-t*	NB-LRR	Rice blast	*Magnaporthe oryzae*	[144]
	*Pi-ta*	NB-LRR	Rice blast	*M. oryzae*	[145]
	*Pi-b*	NB-LRR	Rice blast	*M. oryzae*	[146]
	*Pi-d2*	B-lectin receptor kinase	Rice blast	*M. oryzae*	[146]
	*Pi9*	NB-LRR	Rice blast	*M. oryzae*	[147]
	*RGA 5*	NB-LRR	Rice blast	*M. oryzae*	[148]
	*Xa1*	NB-LRR	Bacterial blight	*X. oryzae*	[149]
	*Xa5*	TFIIA Transcription factor	Bacterial blight	*X. oryzae*	[150]
	*Xa7*	Executer R protein	Bacterial blight	*X. oryzae*	[151]
	*Xa10*	Executer R protein	Bacterial blight	*X. oryzae*	[125]
	*Xa21*	Receptor kinase	Bacterial blight	*X. oryzae*	[152]
	*Xa23*	Executer R protein	Bacterial blight	*X. oryzae*	[126]
	*Xa26*	Receptor kinase	Bacterial blight	*X. oryzae*	[153]
	*Xa27*	No homolog	Bacterial blight	*X. oryzae*	[127]
*Arabidopsis thaliana*	*FLS2*	NB-LRR	Necrosis	*Pseudomonas syringae*	[154]
	*RPM1*	NB-LRR	Necrosis	*Peronospora parasitica*	[155]
	*RSP2*	NB-LRR	Necrosis	*P. syringae*	[156]
Tomato	*Cf-2*	NB-LRR	Leaf mold	*Cladosporium fulvum*	[157]
	*Prf*	NB-LRR	Necrosis	*P. syringae*	[158]

### 3.3. Metagenomic Dynamics

Plants contain a rich diversity of microbial communities that colonize the roots during the life cycle of host plants. These host-associated microbes provide beneficial traits to plants, such as disease suppression, nutrient uptake, and promotion of plant growth [159,160]. Moreover, microbes activate defense mechanisms that increase the host plant’s resilience against pathogens, resulting in significant improvement in disease-combating efficiency [161]. Bacterial antagonists belonging to genera such as Enterobacter, Comamonas, Pantoea, and Microbacterium have shown significant biocontrol against rice blast disease and have stimulated the expression of genes involved in defense responses, such as *OsPAD4*, *OsCEBiP*, *OsEDS1*, and *OsCERK1* in rice seedlings [162]. Similarly, root-associated microbes like *Pantoea* sp. EA106 and *Pseudomonas* sp. EA105 induce disease suppression in *M. oryzae*-inoculated rice plants by triggering ET- and JA-induced systematic resistance [163]. However, advances in molecular biology have led to the development of omics techniques, such as metagenomics, which have recently gained importance in exploring the diversity of plant–microbe interactions in combating disease. Metagenomics has the potential to target numerous unique signature loci in pathogen-affected plants. This approach has already been utilized in agriculture to identify novel genes, enzymes, and microbial communities involved in disease suppression [160,164].

A recent study conducted by Llontop et al. [165] found genes and key bacterial taxa, including 33,000 archaeal and bacterial species, involved in the suppression of fungal pathogens causing root diseases. Similarly, Mendes et al. [166] conducted a metagenomic analysis and showed that *F. oxysporum* infection led to increased microbial diversity, network complexity, and a higher proportion of the genera Flavobacterium, Bacillus, and Dyadobacter in the rhizosphere of the Fox-resistant cultivar compared with the Fox-susceptible cultivar. Moreover, unique functional traits such as the biosynthesis of antifungal genes, rhamnolipids, and phenazines, as well as protein secretion systems, were dominant in the rhizobial community of *P. vulgaris*. In another study, a comparative metagenomic analysis of resistant and susceptible varieties of tomatoes treated with the *R. solanacearum* pathogen revealed the abundant presence of Flavobacterium in the resistant variety. When rhizosphere microbiota from the resistant variety were transplanted into the susceptible variety, reduced disease symptoms were observed compared with non-treated plants Kwak et al. [167]. These studies indicate that microbiome analysis through metagenomics provides a paradigm shift and insights into deciphering the role of the microbial community in protecting against different pathogens. Moreover, the knowledge gained from these aspects through metagenomics could be utilized to engineer rhizosphere or microbial consortia to enhance the plant’s potential to withstand biotic stresses.

## 4. Strategies to Exploit Innate Immune Responses of the Host for Disease Resistance

### 4.1. Introgression of R Genes from Wild Species

The decline in crop diversity has increased the risk of modern crops to pathogen attacks, primarily due to the continuous pursuit of monoculture for high productivity [168]. Landraces and wild species serve as significant resources for novel *R* genes that can be introduced into modern varieties to combat emerging races of pathogens [169]. Hence, there is an imperative need to identify new *R* genes/alleles and to introduce them into modern varieties to improve disease resistance. Indeed, various *R* genes, such as *Xa21*, *Xa23*, *Xa27*, *Yr36*, *CcRpp1*, and *Fhb7*, have been introgressed from landraces or wild species of plants into modern varieties to confer resistance against pathogens [126,127,170]. The *Fhb7* gene, derived from wild relatives of wheat, confers resistance against Fusarium head blight [171], while *CcRpp1*, introduced from a wild pigeon pea, showed strong resistance against soybean rust [172]. Similarly, in another study, the *Rph* gene was introgressed from wild barley to carry resistance to the leaf-rust pathogen *Puccinia hordei* [173]. However, an efficient field-trial platform is required to identify effective elite alleles and *R* genes involved in disease resistance for breeding programs. Natural-based selection for severely damaging crop pathogens must be developed for the widespread screening of germplasm resources to develop resistance. Therefore, plants under high-pressure selection in fields are exposed to continuous infection by pathogens throughout their growth to identify new resistance genes. Similarly, pigm-mediated resistance was confirmed using nursery trials at different locations over multiple years, inoculating with various isolates [174,175].

### 4.2. Identification and Acceleration of R Gene Cloning

The identification and cloning of *R* genes are expensive and slow processes, primarily due to the preparation of libraries and artificial bacterial chromosomes. However, in recent years, advances in bioinformatics and genome sequencing technology have enabled the exploration of genomic regions linked with complex traits such as disease resistance [169]. Mapping-by-sequencing is also gaining importance in identifying and cloning novel *R* genes in plants [176]. Genome-wide association studies (GWAS) have been used to determine the position of *R* genes [177]. For example, *R* genes against rice blast disease, such as *LABR_64* and the partial resistance gene *LABR12*, have been identified using GWAS analysis [54,178]. Similarly, scald-resistance genes, including *Rrs1*, *Rrs2*, *Rrs4*, *Rrs12*, *Rrs13*, *Rrs14*, *Rrs15*, *Rrs17*, and *Rsr18*, have been identified in barley germplasm across different chromosomes using GWAS and mapped through different molecular markers [158,179,180].

Association genetics combined with gene-enrichment sequencing (AgRenSeq) is another powerful tool for identifying NLR-like genes from landraces or wild species. AgRenSeq, which combines RenSeq with association mapping to identify pan-genome variations in diverse crop germplasms, has been used to clone *R* genes such as *SrTA1662*, *Sr33*, and *Sr45* in wheat. Mut-RenSeq, a technique that combines mutagenesis and RenSeq, was used to identify and isolate two resistance genes of stem rust, namely *Sr45* and *Sr22*, in wheat [181]. Similarly, MutChromSeq combines chromosome flow sorting, EMS mutagenesis, and high-throughput sequencing to identify induced mutations in plants by comparing them with parental chromosomes [182]. Gao et al. [183] reported a new method that targets chromosome-based cloning by long-range assembly (TACCA). TACCA assembles complex genomes by combining Chicago long-range linkage with chromosome flow sorting. This technique was employed to clone the *Lr22a* leaf-rust-resistant gene in 4 months using ethyl methylsulfonate and marker information in wheat plants.

### 4.3. CRISPR/Cas9-Mediated Genome Engineering to Confer Disease Resistance

Conventional breeding plays a crucial role in improving plant resistance to pathogens. However, identifying plants with the desired traits from such a vast population is labor-intensive and time-consuming [184]. The rapid advancement of genome-engineering approaches in recent decades has enabled the modification of numerous components of the plant immune system to achieve long-term resistance against pathogens [185,186]. Recently, CRISPR/Cas9 has offered promising prospects for genome engineering to improve plant disease resistance against dominant phytopathogens [187,188,189]. For instance, CRISPR/Cas9 was successfully employed to improve resistance in wheat against powdery mildew by simultaneously targeting three homologs of MLO i.e., TAMLO-A, -B, and -D [190]. In another study, knockout of the *OsERF922* gene increased resistance against the blast fungal pathogen in rice [191]. Moreover, CRISPR/Cas9-targted mutagenesis of the eukaryotic translation initiation factor 4E (eIF4E) resulted in enhanced resistance to potyviruses in *A. thaliana* [192] and cucumber [193]. Similarly, CRISPR/Cas9-mediated engineering of TaNFLX1 has been employed in targeted mutagenesis to confer resistance against *Phytophthora infestans* in wheat. It effectively reduces pathogen proliferation post-infection [194]. In *Brassica napus*, knockout of the *BnWRKY70* gene increases host resistance against *S. sclerotiorum* [22], and CRISPR/Cas9-mediated mutations in three salicylic acid 5 hydroxylase (*OsS5H*) genes showed resistance against *X. oryzae* in *O. sativa* [195].

**Figure 2:**
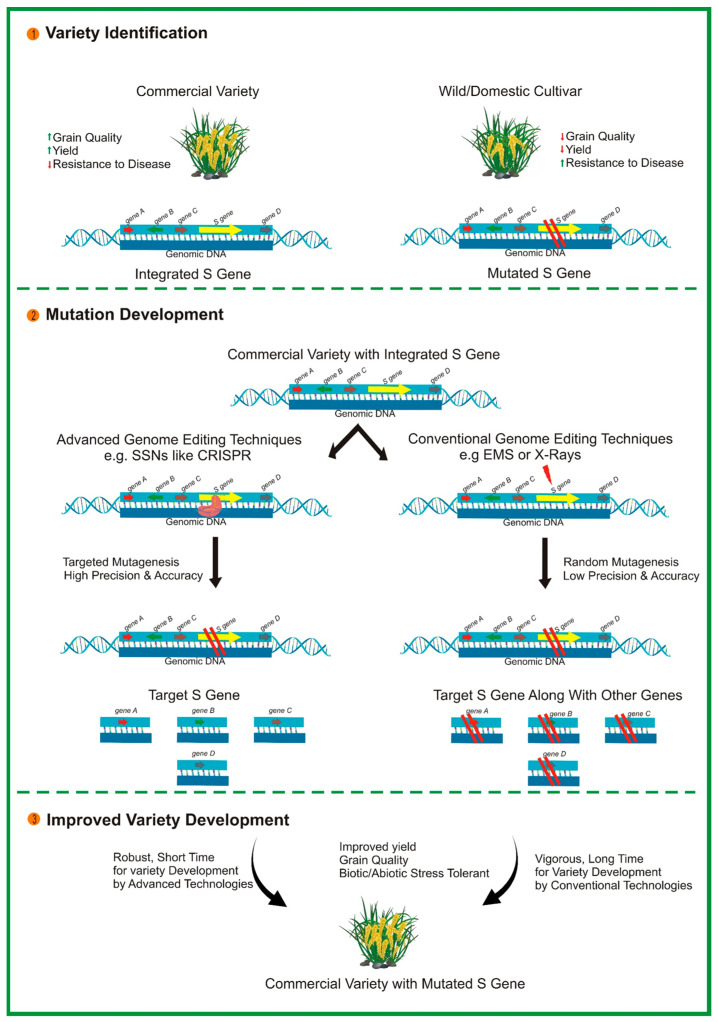
Illustration of the development of disease-resistant varieties in plants by targeting susceptibility (*S*) gene/s via the CRISPR/Cas9 system.

CRISPR/Cas9 also offers significant potential for engineering *S* genes, which are essential for facilitating the infectious process of pathogens (see Figure 2). Inactivating plant *S* genes through genome editing is a novel approach for conferring broad-spectrum disease resistance in various economically important crops [196]. For instance, eIF4E is required for the cellular infection cycle of potyviruses. The 5′-terminal-capped protein interacts with eIF4E to initiate the translation of viral proteins. CRISPR/Cas9-mediated mutagenesis of eIF4E has led to improved resistance to potyviruses in cassava [197]. A mutation in MLO introduced through CRISPR/Cas9 has mediated resistance in tomato [88] and wheat [198] via a non-transgenic system. Furthermore, a mutation introduced in DOWNY MILDEW RESISTANCE 6 (DMR6) through CRISPR/Cas9 in grapevines resulted in increased resistance to downy mildew disease [199]. Overall, the studies mentioned above illustrate the outstanding performance of CRISPR/Cas9 in developing disease-resistant crop varieties. Despite the remarkable achievements to date, challenges such as off-target effects remain to be addressed. However, potential future avenues for this technology in terms of its design and application could be considered. Further identification of *S* genes in various plant species will pave the way for the long-term development of disease resistance using CRISPR/Cas9 technology.

### 4.4. Nanohybrid-Induced Plant Disease Resistance

The emergence of precise and modern genome-engineering techniques, such as CRISPR/Cas9, has significantly increased the versatility of gene editing due to their superior efficacy in inducing targeted mutagenesis in the genome [185,188]. However, these advanced genetic engineering techniques still face various challenges, including modularity, epigenetics, high complexity, standardization, difficulties in characterization, and the risk of accidental release into wild species. Moreover, genetic modification is an irreversible process, raising significant social concerns [200,201].

**Figure 3:**
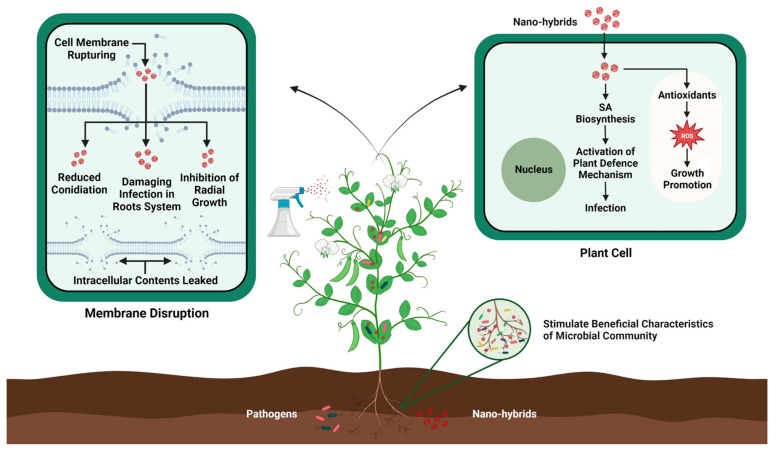
Mechanistic action of nanohybrid-induced pathogen resistance in plants.

Nanohybrids, with their valuable and unique properties, have emerged as a stimulating branch of nanotechnology with a vast variety of applications in the agriculture sector. These nanohybrids endow augmented new properties such as tolerance against environmental stresses, artificial photosynthesis, and improved plant innate immunity against emerging pathogens [202,203]. Moreover, the advent of nanohybrids has also revolutionized the field of plant genetic engineering by enhancing plant resistance against pathogens without causing any harmful effects on the environment (see Figure 3). For example, silver nitrate coated with titanate nanotubes (AgTNTs) significantly inactivates the replication of *Botrytis cinerea* through the photoinactivation method. The morphology and marked cytotoxicity of AgTNT trigger ROS production, which inhibits the further spread of conidia [204]. Similarly, Sidhu et al. [205] reported that MgO–sepiolite nanohybrids showed effective antifungal activity against devastating pathogens such as *F. fujikuroi*, *F. verticillioides*, and *Bipolaris oryzae* compared with MgO NP-treated plants. MgO–sepiolite causes extensive damage to hyphae by breaking the cell wall and inducing internal damage. Silver–platinum nanohybrids (AgPtNHs) produced using a *Dioscorea bulbifera* tuber extract exhibited strong antibacterial activity against *Staphylococcus aureus* and *Pseudomonas aeruginosa*, as well as significantly inhibited biofilm formation [206], and chitosan-based nanohybrids loaded with prothioconazole exhibited significant antifungal activity against wheat scab disease and improved plant growth compared with control plants [207]. However, as information on nanohybrid-based genome engineering is limited, further research in this area is necessary to extend our knowledge. Moreover, the rapid advancement of nanotechnology in recent decades has redirected plant genetic engineering toward nanoparticle-mediated gene transformation to overcome these challenges [208]. For instance, Hajiahmadi et al. [209] used silicon nanoparticles (SiNPs) to deliver the *cryIAb*-gene-containing plasmid into tomato plants by injecting the solution into the abaxial surface of tomato leaves. Subsequent bioassays and molecular analyses confirmed the expression of *cryIAb*, resulting in increased resistance of tomato plants against *Tuta absoluta*. Similarly, chitosan nanoparticles (chitosan NPs) have been shown to enhance cargo delivery, resulting in a significant increase in protection or durable resistance against pearl millet downy mildew in pearl millet [210]. Although nanohybrids provide a cost-effective and reliable approach for gene transformation in plants, the widespread use of nanotechnology nevertheless raises concerns regarding its potential adverse effects on human health. Nanohybrids can accumulate in microbial and plant systems, thus entering the food chain to affect human health. Therefore, we should focus on the development of biocompatible and biodegradable nanohybrids to reduce their accumulation in the agricultural system.

## 5. Challenges and Future Directions

Modern agricultural practices cause environmental degradation, land pollution, and ecosystem disruption, which are aggravated by the extensive use of chemical fertilizers. This unsustainable scenario in the food and agriculture sector requires innovative solutions. Recent research has unveiled the potential of nanohybrids to enhance disease resistance, improve agricultural inputs, and address agriculture-related challenges, thereby promoting plant resistance and food security. The prospective use of nanoscale agrochemicals, such as nanohybrid-based fertilizers, has transformed traditional agricultural practices, making them more sustainable and efficient. However, the application of nanoproducts in real-life scenarios raises concerns about exposure levels and the potential toxicological effects on human health and the environment. For instance, “Starlink” maize, engineered to express Cry9c, was approved for industrial use and animal feed in the USA but not for human consumption due to its elevated protein levels and potential interactions with the immune system [211]. Further concerns have been raised about the potential horizontal transfer of antibiotic-resistance marker genes to humans and gut bacteria in animals [212]. Therefore, the development of proper testing methods is imperative to evaluate the safety of genetically modified crops and to sustain the agricultural food production system in the future.

Moreover, to mitigate these risks, governmental and non-governmental organizations must implement regulatory frameworks to assess the ecotoxicological effects of genetically modified food products and nano-agrochemicals before their use in any sector. Moreover, addressing the commercial potential, implementation barriers, and policy regulations about the use of nano-agrochemicals in agriculture is imperative to ensure their safe and effective integration into agricultural practices.

## 6. Conclusions

The knowledge of plant–pathogen interactions has experienced exponential growth in recent decades, boosted by significant advancements in computational power and the emergence of molecular technologies. From the identification of PAMPs to the implementation of robust defense mechanisms by plants, the ongoing arms race between hosts and pathogens underscores the intricate nature of these relationships. Furthermore, with the onset of climate change and evolving agricultural practices, new pathogen strains are emerging, requiring the urgent development of sustainable agricultural systems to address these challenges. By delving into the molecular mechanisms underlying plant–pathogen interactions, researchers can uncover fundamental biological processes that offer promising avenues for bolstering crop resilience against diseases. Leveraging this knowledge presents opportunities to devise innovative targeted strategies for crop protection that are both environmentally sustainable and economically viable.

## Data Availability

Not applicable.
